# The Impact of Obesity on Patient-Reported Outcomes Following Two-Level Cervical Disc Replacement

**DOI:** 10.3390/jcm15166407

**Published:** 2026-08-19

**Authors:** Sloane O. Ward, Puranjay Gupta, Shreya Kurup, Maximillian Y. Lee, Dipankar Yettapu, Rohit Rajput, Amy Truong, Madeline Sandberg, Jonah Dujovny, Aditya S. Mazmudar, Arash J. Sayari, Daniel K. Park, Kern Singh

**Affiliations:** 1 Department of Orthopaedic Surgery, Rush University Medical Center, 1611 W. Harrison St. Suite #300, Chicago, IL 60612, USA; 2 Case Western Reserve University School of Medicine, Cleveland, OH 44106, USA; 3 Department of Orthopedics, MedStar Georgetown University Hospital, Washington, DC 20007, USA

**Keywords:** cervical disc replacement, patient-reported outcomes, spine, orthopaedic surgery

## Abstract

**Background:** Body mass index (BMI) is an important consideration in perioperative evaluation. High BMI is correlated with more comorbidities and poorer operative outcomes; however, there is limited literature on the impact of BMI on patient-reported outcome measures (PROMs) after multilevel cervical disc replacement (CDR) surgery. **Objective:** The objective was to evaluate the relationship between BMI and PROMs in two-level CDR. **Methods:** A single-surgeon database was retrospectively reviewed for patients who underwent two-level CDR between August 2017 and October 2024. Patients were stratified as non-obese (BMI < 30 kg/m^2^; n = 44) or obese (BMI ≥ 30 kg/m^2^; n = 34). The final analytic cohort included 78 patients. Mean follow-up was 7.65 ± 5.53 months. Multivariable linear and logistic regression models adjusted for age, hypertension, diabetes, and Charlson Comorbidity Index. Patient-reported outcome measures (PROMs) and minimal clinically important difference (MCID) were also analyzed. Statistical analysis was conducted using Stata 18.0 (StataCorp LP, College Station, TX, USA). **Results:** Obese patients had a higher CCI than non-obese patients (0.90 ± 1.06, 1.87 ± 1.31, *p* = 0.001). Patient-reported outcomes were collected and analyzed after controlling for hypertension, diabetes, CCI scores, and age. At the final postoperative follow-up, only VR12-MCS differed, with the obese cohort having worse scores (60.7 ± 5.0 versus 53.9 ± 10.2, *p* = 0.040). MCID achievement rates did not differ between groups across PROMs (*p* > 0.183 for all). **Conclusion:** Patients with and without obesity demonstrated comparable early-to-midterm PROM trajectories and MCID achievement following two-level CDR. These findings suggest that obesity was not associated with substantially different patient-reported recovery during the observed follow-up period.

## 1. Introduction

Multiple data points are used to evaluate a patient’s potential for achieving successful outcomes after spine surgery. One important factor is body mass index (BMI), as obesity can impact all aspects of surgery. Excess tissue may make dissection more difficult and obscure visualization and identification of critical structures during the approach. Barrie et al. found that obese patients spend more time under anesthesia and have higher estimated blood loss, which can also impair operative field visualization and make device placement more challenging in CDR [1].

BMI’s impact extends postoperatively, where the same study found that more obese patients were discharged to a facility versus home (*p* < 0.05), and had worse postoperative Oswestry Disability Index (ODI) scores than the non-obese cohort [1]. These outcomes may be linked to worse postoperative improvement and increased costs. Naik et al. found that patients with Class II and III obesity undergoing one-level cervical surgery had worse odds of achieving optimal modified Japanese Orthopaedic Association scores (JOA) three (OR 0.8, *p* = 0.01; OR 0.68, *p* = 0.001) and 12 months (OR = 0.82, *p* = 0.03; OR = 0.79, *p* = 0.03) after surgery [2]. For adults, the Centers for Disease Control and Prevention (CDC) BMI classifications define underweight as BMI < 18.5 kg/m^2^, normal weight as 18.5–24.9 kg/m^2^, overweight as 25.0–29.9 kg/m^2^, and obesity as BMI ≥ 30 kg/m^2^. Obesity is further classified as class I (30.0–34.9 kg/m^2^), class II (35.0–39.9 kg/m^2^), and class III (≥40 kg/m^2^). In the present study, BMI was analyzed as a binary variable using the standard obesity threshold of 30 kg/m^2^ [3].

Cervical disc replacement (CDR) is an increasingly utilized treatment option for degenerative disc disease, radiculopathy, and myelopathy, with an increasing body of evidence supporting its use. Prospective randomized studies have also established favorable outcomes after two-level CDR. Davis et al. reported sustained clinical improvement through 4 years after two-level Mobi-C arthroplasty, while Gornet et al. demonstrated sustained improvements in disability, pain, overall success, and segmental motion through 10 years after two-level Prestige LP arthroplasty [4,5]. In their prospective randomized control trial comparing CDR and anterior cervical discectomy and fusion (ACDF), Foley et al. reported lower adjacent segment disease grades (*p* < 0.005) and reduced development of adjacent segment ossification (*p* < 0.001) 20 years after index surgery [6]. The same study also found better total cervical range of motion (47.8° versus 33.4°, *p* = 0.005) and maintenance of range of motion at the 20-year postoperative timepoint (45.6° versus 47.4°, *p* = 0.772) [6]. Chen et al., in their systematic review and meta-analysis of eight studies, found CDR was associated with lower reoperation rates (OR 0.254, *p* < 0.001) and better Visual Analog Score (VAS) Arm scores at final follow-up (OR 0.236, *p* = 0.005) compared to ACDF [7]. Furthermore, Liu et al. found higher BMI to be a risk factor for dysphagia onset within two days of anterior cervical surgery [8]. As more evidence supports the advantages of CDR on top of its motion-preserving benefit, more patients may potentially benefit. However, as obesity prevalence remains high in the United States, a clear understanding of the risk profile of CDR in this patient population is important for patients and surgeons alike.

Prior obesity-related CDR studies have primarily examined perioperative complications or broader single-level and mixed-level cohorts. Berry et al. examined obesity-related perioperative complications after CDR but did not evaluate longitudinal PROM trajectories [9]. Anwar et al. evaluated PROMs and MCID achievement after CDR using the same binary BMI classification of <30 versus ≥30 kg/m^2^ and found postoperative improvement in both groups, with no significant adjusted differences in PROM improvement or MCID achievement, but did not specifically focus on a two-level CDR cohort [10]. Therefore, the relationship between obesity and patient-reported recovery after two-level CDR remains insufficiently characterized. Accordingly, this study evaluated the association between obesity and short- to intermediate-term PROMs and MCID achievement following two-level CDR.

## 2. Methods

### 2.1. Patient Population

**Figure 1:**
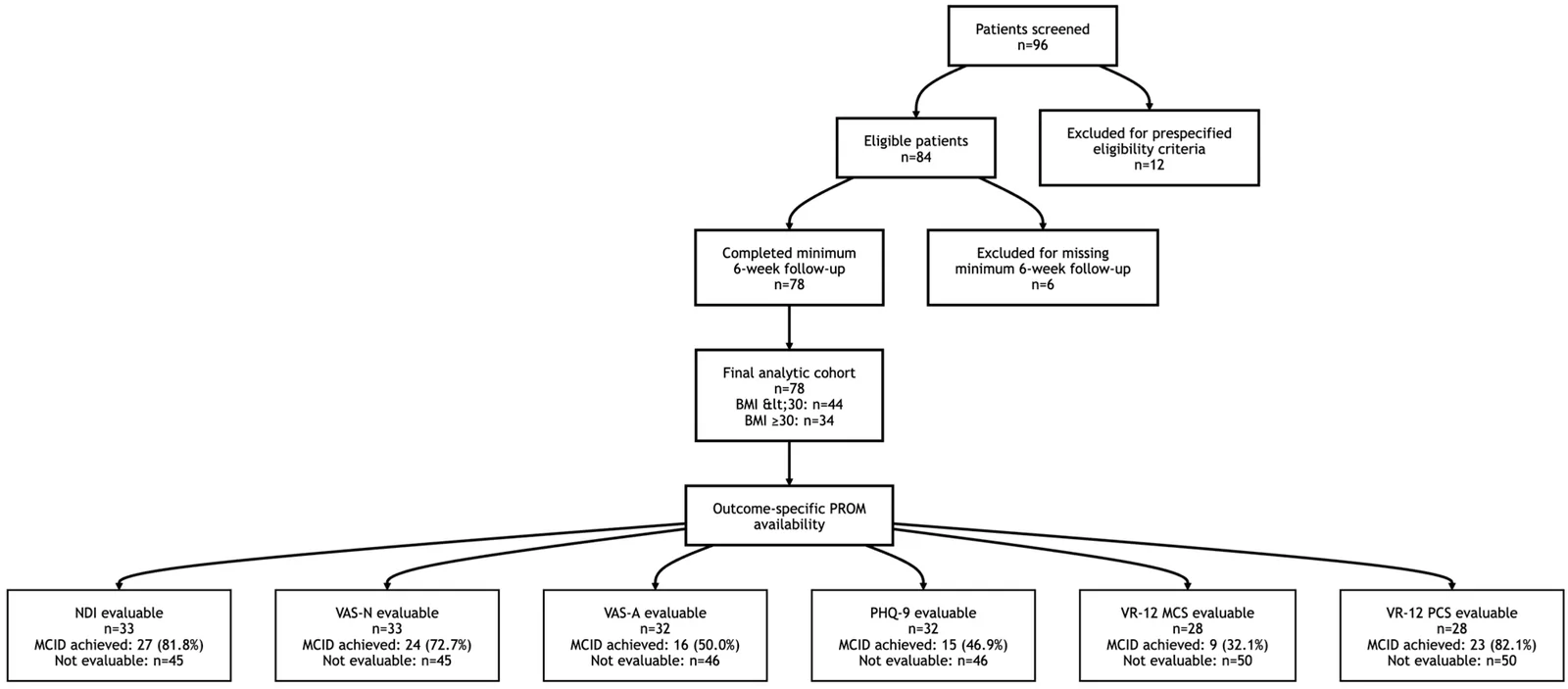
Patient flow and outcome-specific PROM availability. Of 96 screened patients, 78 were included in the final analysis. Patients were classified as non-obese (BMI < 30 kg/m^2^; n = 44) or obese (BMI ≥ 30 kg/m^2^; n = 34). Outcome-specific PROM denominators are shown.

A single surgeon’s database was retrospectively reviewed for patients with documented preoperative BMI undergoing two-level CDR between August 2017 and October 2024 (IRB exemption was granted on 12 December 2023). Patients were excluded if they underwent revision or non-elective CDR, or if the procedure was performed for malignancy, trauma, or infection. Of 96 screened patients, 12 were excluded based on prespecified eligibility criteria. Of the remaining 84 patients, 6 were excluded because they lacked the minimum 6-week postoperative follow-up, leaving 78 patients in the final analytic cohort. Mean follow-up duration was 7.65 ± 5.53 months. PROM availability varied by instrument. Outcome-specific MCID-evaluable denominators were 33 for NDI, 33 for VAS-N, 32 for VAS-A, 32 for PHQ-9, 28 for VR-12 MCS, and 28 for VR-12 PCS (Figure 1).

### 2.2. Data Collection

Gender, age, BMI, ethnicity, insurance type, comorbidities, ASA score, and Charlson Comorbidity Index (CCI) scores were obtained (Table 1). Levels of decompression, estimated blood loss (EBL), length of stay (LOS), and narcotic consumption in oral morphine equivalents (OME) were also collected (Table 2). Patient-reported outcome measures (PROMs) were also evaluated through Visual Analog Scale Neck and Arm (VAS-N/A) scores, Patient Health Questionnaire (PHQ-9), Neck Disability Index (NDI), Veterans RAND-12 Mental Composite Score (VR-12 MCS), and Veterans RAND-12 Physical Composite Scores (VR-12 PCS) (Table 3).

### 2.3. Statistical Analysis

Patients were stratified into non-obese (BMI < 30 kg/m^2^) and obese (BMI ≥ 30 kg/m^2^) cohorts. Baseline continuous variables were compared using independent-samples t tests, while categorical variables were compared using chi-square or Fisher’s exact tests, as appropriate.

Separate multivariable linear regression models were used to evaluate continuous PROM scores at 6 weeks postoperatively, at final postoperative follow-up, and to estimate changes from baseline at each postoperative time point. The dependent variables were NDI, VAS-Neck, VAS-Arm, PHQ-9, VR-12 MCS, and VR-12 PCS scores. The primary independent variable was the BMI group. All models were adjusted for age, hypertension, diabetes, and CCI score. The number of patients included in each model varied according to outcome-specific PROM availability and is reported for each PROM and time point.

MCID achievement was analyzed as a binary outcome using multivariable logistic regression. Patients were classified as having achieved or not achieved MCID according to previously established thresholds. Logistic regression models included BMI group, age, hypertension, diabetes, and CCI score as covariates. MCID analyses were restricted to patients with the required preoperative and postoperative PROM data, and outcome-specific denominators are reported. Statistical analysis was conducted using Stata 18.0 (StataCorp LP, College Station, TX, USA). MCID thresholds were prespecified using previously published cervical spine and spine-surgery literature. The thresholds were 8.5 points for the Neck Disability Index, 2.6 points for VAS-Neck, 4.1 points for VAS-Arm, 3.0 points for the PHQ-9, 4.1 points for the VR-12 Physical Component Score, and 11.2 points for the VR-12 Mental Component Score [11,12,13,14,15].

Improvement was defined as a decrease in NDI, VAS-Neck, VAS-Arm, or PHQ-9 scores and an increase in VR-12 PCS or VR-12 MCS scores. MCID achievement was evaluated separately at 6 weeks, 12 weeks, and final follow-up, with final follow-up defined as the most recent available assessment at 6 months, 1 year, or 2 years. Overall MCID achievement was defined as meeting the applicable threshold at any postoperative assessment. PROM analyses used complete-case data at each postoperative time point. Patients were included in a given analysis only when the relevant PROM was available at that time point. Change-score and MCID analyses required the corresponding preoperative and postoperative scores. No imputation was performed for missing PROM data.

Because this was a retrospective analysis of all eligible patients within the study period, no a priori sample size or power calculation was performed. The analyses were considered exploratory and hypothesis-generating. Effect estimates and 95% confidence intervals were used to characterize the magnitude and precision of observed associations.

## 3. Results

### 3.1. Descriptive Analysis

Seventy-eight patients met inclusion criteria. Forty-four were non-obese (BMI < 30), and 34 patients were obese (BMI ≥ 30). The average BMI was 25.32 ± 2.59 in the non-obese cohort, and 33.70 ± 3.80 in the Obese cohort (*p* < 0.001). Patients in the obese cohort were older than the non-obese cohort (47.89 ± 10.30, 52.93 ± 8.37 years old, *p* = 0.023). The cohorts had significantly different comorbidity burdens. Obese patients had a higher CCI than non-obese patients (0.90 ± 1.06, 1.87 ± 1.31, *p* = 0.001). Most patients meeting inclusion criteria were male (65.38%), White (78.08%), and had private health insurance (79.41%). ASA scores did not differ between cohorts (1.89 ± 0.46 versus 2.14 ± 0.65, *p* = 0.073). Operative time, EBL, and LOS did not differ significantly between cohorts (*p* ≥ 0.440 for all).

### 3.2. Primary Outcome Measures

Patient-reported outcomes were collected and analyzed after controlling for hypertension, diabetes, CCI scores, and age. At baseline, no statistically significant between-group differences were detected in NDI, VAS-N, VAS-A, PHQ-9, or VR-12 PCS scores (*p* ≥ 0.389 for all comparisons). However, VR-12 MCS scores were significantly inferior for the Obese cohort before surgery (56.5 ± 8.0 versus 47.1 ± 13.7, *p* = 0.049). Six weeks after surgery, the obese group continued to have lower VR-12 MCS scores (60.4 ± 7.3 versus 52.1 ± 8.9, *p* = 0.031). At the final postoperative follow-up, VR12-MCS continued to differ, with the obese cohort having worse scores (60.7 ± 5.0 versus 53.9 ± 10.2, *p* = 0.040). No statistically significant between-group differences were detected in changes in NDI, VAS-N, VAS-A, PHQ-9, or VR-12 PCS scores at the evaluated time points (*p* > 0.072 for all, Table 4). There were also no statistically significant between-group differences in MCID achievement rates across PROMs (*p* > 0.183 for all comparisons).

## 4. Discussion

This study evaluated whether obesity was associated with patient-reported outcomes following two-level cervical disc replacement. Among 78 patients, those with obesity were older, had a greater comorbidity burden, and reported lower baseline VR-12 MCS scores than non-obese patients. After adjustment for the corresponding baseline PROM, age, hypertension, diabetes, and Charlson Comorbidity Index score, no statistically significant between-group differences were detected in NDI, VAS-Neck, VAS-Arm, PHQ-9, or VR-12 PCS scores at the evaluated postoperative time points. MCID achievement rates also did not differ significantly between groups. In contrast, VR-12 MCS scores remained lower in the obese cohort at 6-week and final postoperative follow-up. These findings suggest that obesity was not associated with statistically detectable differences in most evaluated PROMs after two-level CDR.

Age and higher BMI can play a role in worsening cervical spine degeneration. Kocur et al. studied the relationship between age, BMI, and superficial neck muscle stiffness in women and found that advanced age and higher BMI significantly reduced the craniovertebral angle, possibly contributing to worsening cervical degenerative disc disease [16]. Adipose tissue in the neck may also play a role in degenerative disease. Emir and Emir found that women with cervical disc degeneration had thicker subcutaneous fat (*p* < 0.001) [17]. Thus, higher BMI patients may be more apt to have degenerative disease requiring operative intervention.

Moreover, patients in the higher BMI cohort presented with higher rates of hypertension and diabetes, which contribute to the higher CCI scores observed in the group. Numerous prior studies have found a strong association between elevated BMI scores and comorbidities such as hypertension and diabetes [18,19]. Khan et al. found that a higher mortality risk seen in patients is greatly mediated by complications that are associated with diabetes, such as myocardial infarction [20]. Additionally, obesity in males contributes to an increased risk of myocardial infarction [21]. While the current study is consistent with previous evidence of greater age, comorbidities, and CCI scores in obese populations, our findings underscore that these patients can achieve comparable PROMs as their non-obese counterparts following surgery.

High BMI may increase perioperative risks after cervical disc replacement. Subramanian et al. analyzed 2005–2020 ACS-NSQIP data from 5397 patients undergoing primary one- or two-level CDR. Patients were categorized as nonobese (BMI 18.5–29.9 kg/m^2^), class I obesity (BMI 30–34.9 kg/m^2^), or class II/III obesity (BMI ≥ 35 kg/m^2^). Class II/III obesity was independently associated with increased 30-day readmission (OR 3.32, *p* = 0.002) and nonhome discharge (OR 2.51, *p* = 0.027). Neither 30-day reoperation nor postoperative morbidity differed significantly between groups [22]. Though obesity and high BMI are often considered major risk factors for surgical site infection and thromboembolism, Subramanian et al. did not find these relationships in their retrospective database study. They did not find a difference between BMI groups in superficial wound infections (*p* = 0.589), deep incisional infections (*p* = 0.374), wound disruptions (*p* = 0.091), pulmonary embolisms (*p* = 1), or ventilator requirement over 48 h (*p* = 1). Their work provides good insight into postoperative risks in CDR based on BMI, but they did not investigate BMI’s relationship with PROMs as the current study does.

Singh et al. retrospectively reviewed the PearlDiver database (PearlDiver Technologies, Colorado Springs, CO, USA) for adults who underwent single-level CDR between 2010 and October 2022 [23]. They categorized 12,545 patients into four groups: healthy weight (BMI < 25), overweight (BMI 25–30), obese (BMI 30–40), and morbidly obese (BMI > 40). After matching for age, sex, and CCI, they found no differences in infections (*p* = 0.267), wound dehiscence (*p* = 0.445), or pulmonary embolism (*p* = 0.571) during the 90-day postoperative period. Two years after surgery, they found no difference in dysphagia (*p* = 0.168), dysphonia (*p* = 0.698), or implant failure (*p* = 0.772). The healthy weight cohort did have more anterior revision surgeries, however (*p* = 0.005). Singh et al.’s work provides valuable insight into the impact of BMI on single-level CDR. Our current paper builds on their work by investigating BMI in a two-level CDR.

The aim of the study was to determine whether there are variations in patient-reported outcomes following two-level CDR surgery based on BMI. Interestingly, baseline reporting for the non-obese and obese cohorts was comparable across all PROMs except VR-12 MCS scores. The VR-12 items, which have both physical and mental components, include questions addressing restrictions related to mental health, pain, energy, social functioning, and general health [24]. A prior study by Nie et al. studied the impact of poor VR-12 MCS baseline scores in patients undergoing cervical disc replacement surgery and reported inferior results in mental health, physical function, pain, and disability outcomes throughout the recovery of the subgroup of patients [25]. Moreover, the previous literature has established associations between mental health measures, including the PHQ-9 and VR-12 MCS, following cervical spine procedures [26]. In the current study, PHQ-9 scores were included along with SF-12 MCS scores. Although PHQ-9 scores were higher in the obese cohort preoperatively and at all postoperative time points, the differences were not statistically significant compared with their non-obese counterparts. This suggests that VR-12 MCS scores may be more sensitive for detecting role limitations from mental health conditions than the PHQ-9. The findings may also be associated with the wording of the questionnaires. Schalet et al. compared PROMIS Global Mental Health instruments with the VR-12 MCS questionnaire and found differences in questions referencing mental health and quality of life [24]. VR-12 MCS differs in that there are two questions on how emotional issues in patients could interfere with productivity, which is not mentioned in PROMIS global mental health instruments [24]. Additional studies should examine the PHQ-9 scoring framework to determine differences in question phrasing compared with the VR-12 MCS, to solidify the findings established in the current study.

VAS-A, VAS-N, and NDI scores showed no statistically significant differences between the two cohorts. Although prior studies have described clinical improvements after one- and two-level cervical disc replacement [27], our findings suggest that similar short- to intermediate-term PROM patterns were observed among obese and non-obese patients in this selected cohort. Further, in a study examining patients undergoing multilevel CDR, mean VAS-N and NDI scores decreased from baseline to seven years postoperatively [28]. Therefore, these findings describe potential observed improvements in neck pain and disability among both obese and non-obese patients following two-level CDR.

### Limitations

Our study is not without limitations. First, the retrospective design of this study may introduce selection bias in the patient cohort. This was mitigated to the extent possible by establishing set inclusion and exclusion criteria before interrogating the database. Second, the final postoperative follow-up varied, with a mean of 7.65 ± 5.53 months. The short average follow-up time in this study may not have provided sufficient time to elucidate differences in PROM between cohorts. The known intraoperative challenges associated with higher BMI may not affect PROMs less than 1 year postoperatively. The increased EBL and adipose tissue during surgery in obese patients require more retraction to maintain a clear working space. This additional stress on soft tissue can cause more postoperative soft tissue edema, potentially leading to dysphagia and dysphonia. More adipose tissue in obese patients also makes device position evaluation on imaging difficult, especially intraoperatively, where soft-tissue swelling can compound visualization difficulties. Obese patients also have more compromised airways at baseline, and obese patients are more likely to have obstructive sleep apnea, which further reduces baseline airway function. These additional perioperative challenges may create outcome differences that cannot be fully elucidated in the short follow-up period of this study. Future studies should analyze PROMs with longer follow-up to gain a more comprehensive view of outcomes in obese patients. Investigating additional PROMs related to laryngo- and pharyngo-esophageal function, which this study did not do, would also be additive. Third, this is a retrospective analysis using a single surgeon’s experience database. Future studies involving more surgeons can increase the generalizability of these results. Moreover, obesity is linked to other complications that the current study was not powered to evaluate. For example, the previous literature studying the effects of obesity on spine surgery found obesity to be associated with elevated rates of surgical site infection and venous thromboembolism [26]. Clinicians should inform patients to optimize their health to minimize additional risks associated with obesity when undergoing cervical spine surgery. Fourth, although PROMs were collected at multiple postoperative time points, the repeated measurements were analyzed separately at prespecified time points rather than with a longitudinal mixed-effects model or generalized estimating equation. Therefore, within-patient correlation across repeated PROM assessments was not directly modeled. A longitudinal model may have been difficult to estimate reliably because the outcome-specific analytic populations were small, ranging from 28 to 33 patients, and the timing and availability of final PROM assessments varied between patients. Additionally, as no formal equivalence or noninferiority analysis was performed, nonsignificant between-group comparisons should not be interpreted as evidence that obese and non-obese patients had equivalent outcomes. The small outcome-specific sample sizes and corresponding confidence intervals also limit the ability to exclude clinically meaningful differences between cohorts. Future studies with larger cohorts and more complete, consistently timed follow-up should use longitudinal modeling to further evaluate PROM trajectories after two-level CDR.

## 5. Conclusions

Among patients undergoing two-level CDR, those with and without obesity demonstrated comparable early-to-midterm PROM trajectories and MCID achievement. Within the limitations of this retrospective cohort, obesity was not associated with substantially different patient-reported recovery during the available follow-up period. Our findings suggest that two-level CDR may be an effective treatment in appropriately selected obese patients, and warrant confirmation in larger prospective studies with longer follow-up and appropriate comparative groups.

## Figures and Tables

**Table 1:** Patient Demographics.

Characteristic	Total (n = 78)	Non-Obese (n = 44)	Obese (n = 34)	* *p*-Value
Age (mean ± SD, years)	50.09 ± 9.78	47.89 ± 10.30	52.93 ± 8.37	**0.023**
Male Gender	65.38% (51)	65.91% (29)	64.71% (22)	0.912
BMI (mean ± SD, kg/m^2^)	28.58 ± 5.14	25.32 ± 2.59	33.70 ± 3.80	**<0.001**
*Ethnicity*				0.096
Asian	4.11% (3)	5.00% (2)	3.03% (1)	
African American	6.85% (5)	0% (0)	15.15% (5)	
Hispanic	9.59% (7)	12.50% (5)	6.06% (2)	
White	78.08% (57)	80.00% (32)	75.76% (25)	
Other	1.37% (1)	2.50% (1)	0% (0)	
*Comorbidities*				
Smoker	2.63% (2)	2.38% (1)	2.94% (1)	0.879
Hypertension	21.05% (16)	7.14% (3)	38.24% (13)	**0.001**
Diabetes	8.97% (7)	2.27% (1)	17.65% (6)	**0.018**
ASA Score (mean ± SD)	2.00 ± 0.56	1.89 ± 0.46	2.14 ± 0.65	0.073
CCI Score (mean ± SD)	1.32 ± 1.22	0.90 ± 1.06	1.87 ± 1.31	**0.001**
*Insurance Type*				0.212
Medicare/Medicaid	3.95% (3)	2.38% (1)	5.88% (2)	
Workers’ Comp	23.68% (18)	30.95% (13)	14.71% (5)	
Private	72.37% (55)	66.67% (28)	79.41% (27)	

BMI = Body Mass Index; ASA = American Society of Anesthesiologists; CCI = Charlson Comorbidity Index; SD = Standard Deviations; Workers’ Comp = workers’ compensation. * *p*-value calculated using chi-square tests for categorical variables or independent samples *t*-test for continuous variables. **Boldface** indicates significance.

**Table 2:** Perioperative Characteristics.

Characteristic	Total (n = 78)	Non-Obese (n = 44)	Obese (n = 34)	* *p*-Value
Operative Time (min)				
Mean ± SD	59.71 ± 10.71	58.92 ± 11.33	60.79 ± 9.90	0.489
Estimated Blood Loss (mL)				
Mean ± SD	26.69 ± 6.32	26.19 ± 5.39	27.34 ± 7.40	0.440
Postoperative Length of Stay (hours)				
Mean ± SD	6.49 ± 3.55	6.30 ± 3.79	6.77 ± 3.22	0.606
*Spinal Pathology*				
Foraminal Stenosis	41.03% (32)	40.91% (18)	41.18% (14)	0.515
Central Stenosis	80.26% (61)	79.07% (34)	81.82% (27)	0.765
Herniated Nucleus Pulposus	97.44% (76)	97.73% (43)	97.06% (33)	0.853
Myeloradiculopathy	89.74% (70)	86.38% (38)	94.12% (32)	0.587
POD 0 VAS Pain	4.98 ± 2.34	5.16 ± 2.35	4.74 ± 2.35	0.478
POD 0 Narcotic Consumption (OME)	19.66 ± 16.73	20.43 ± 18.67	18.56 ± 13.81	0.661

POD = Postoperative Day of Discharge; SD = Standard Deviation; VAS = Visual Analog Scale; OME = Oral Morphine Equivalents; * *p*-value calculated using chi-square tests for categorical variables or independent samples *t*-tests for continuous variables.

**Table 3:** Patient-Reported Outcome Measures and Minimum Clinically Important Difference.

	Total (n = 78)	Non-Obese (n = 44)	Obese (n = 34)	* *p*-Value
*Pre-Op*				
NDI	38.3 ± 19.1	37.8 ± 21.7	38.8 ± 15.8	0.653
VAS-N	6.3 ± 2.5	6.5 ± 2.8	6.1 ± 2.1	0.602
VAS-A	5.5 ± 2.9	5.5 ± 2.8	5.4 ± 3.0	0.768
PHQ-9	5.8 ± 5.1	5.3 ± 5.0	6.5 ± 5.4	0.389
VR-12 MCS	52.4 ± 11.7	56.5 ± 8.0	47.1 ± 13.7	**0.049**
VR-12 PCS	36.1 ± 9.8	37.0 ± 10.2	35.0 ± 9.5	0.719
*6-week Post-Op*				
NDI	21.0 ± 17.0	19.2 ± 19.2	23.0 ± 14.8	0.583
VAS-N	2.3 ± 2.1	2.3 ± 2.3	2.3 ± 1.9	0.551
VAS-A	1.8 ± 2.5	0.9 ± 1.8	2.7 ± 2.9	0.072
PHQ-9	3.0 ± 3.6	2.2 ± 2.8	4.2 ± 4.5	0.434
VR-12 MCS	56.8 ± 8.9	60.4 ± 7.3	52.1 ± 8.9	**0.031**
VR-12 PCS	45.1 ± 11.4	47.2 ± 10.2	42.4 ± 12.8	0.695
*Final Post-Op*				
NDI	14.2 ± 14.4 (n = 33)	11.7 ± 12.2 (n = 17)	17.2 ± 16.5 (n = 16)	0.454
VAS-N	1.8 ± 2.1 (n = 33)	1.7 ± 1.8 (n = 17)	1.9 ± 2.4 (n = 16)	0.853
VAS-A	1.4 ± 2.2 (n = 32)	0.8 ± 1.5 (n = 17)	2.1 ± 2.8 (n = 15)	0.341
PHQ-9	2.6 ± 3.5 (n = 32)	1.5 ± 2.2 (n = 17)	3.8 ± 4.4 (n = 15)	0.181
VR-12 MCS	57.7 ± 8.3 (n = 28)	60.7 ± 5.0 (n = 16)	53.9 ± 10.2 (n = 12)	**0.040**
VR-12 PCS	49.2 ± 10.6 (n = 28)	52.4 ± 5.6 (n = 16)	45.0 ± 13.9 (n = 12)	0.171
*Δ Pre-Op to 6-week Post-Op*				
NDI	16.1 ± 19.8	12.8 ± 22.5	19.5 ± 17.0	0.490
VAS-N	3.7 ± 2.9	3.4 ± 3.0	4.0 ± 2.9	0.587
VAS-A	3.3 ± 3.2	4.0 ± 3.2	2.7 ± 3.3	0.329
PHQ-9	2.7 ± 4.5	1.8 ± 3.9	3.9 ± 5.2	0.839
VR-12 MCS	−5.0 ± 13.5	−2.6 ± 6.1	−8.2 ± 19.9	0.416
VR-12 PCS	−7.8 ± 10.9	−9.2 ± 6.9	−5.9 ± 15.2	0.902
*Δ Pre-Op to Final Post-Op*				
NDI	25.1 ± 20.2	27.8 ± 18.1	22.3 ± 22.4	0.684
VAS-N	4.5 ± 2.7	4.9 ± 2.5	4.1 ± 2.9	0.453
VAS-A	3.9 ± 3.2	4.7 ± 2.7	3.1 ± 3.6	0.671
PHQ-9	2.8 ± 4.6	2.6 ± 3.5	3.1 ± 5.7	0.838
VR-12 MCS	−4.9 ± 12.0	−2.4 ± 8.1	−8.2 ± 15.6	0.476
VR-12 PCS	−12.0 ± 10.3	−13.1 ± 7.4	−10.5 ± 13.4	0.764
*MCID Achievement*				
NDI	81.8% (27/33)	82.4% (14/17)	81.3% (13/16)	0.885
VAS-N	72.7% (24/33)	82.4% (14/17)	62.5% (10/16)	0.183
VAS-A	50.0% (16/32)	52.9% (9/17)	46.7% (7/15)	0.583
PHQ-9	46.9% (15/32)	47.1% (8/17)	46.7% (7/15)	0.924
VR-12 MCS	32.1% (9/28)	25.0% (4/16)	41.7% (5/12)	0.356
VR-12 PCS	82.1% (23/28)	87.5% (14/16)	75.0% (9/12)	0.793

* *p*-value calculated using Student’s *t*-test for continuous variables at the preoperative time point. At postoperative time points and ∆PROMs, *p*-values were calculated using multivariable linear regression and logistic regression accounting for hypertension, diabetes, Charlson Comorbidity Index scores, and age. VAS-N = Visual Analog Scale-Neck Pain; VAS-A = VAS-Arm Pain; NDI = Neck Disability Index; PHQ-9 = 9-Item Patient Health Questionnaire; VR-12 MCS = Veterans RAND-12 Mental Composite Score; VR-12 PCS = Veterans RAND-12 Physical Composite Score. Δ represents the preoperative score minus the postoperative score. For NDI, VAS-Neck, VAS-Arm, and PHQ-9, lower scores indicate improvement; therefore, positive Δ values indicate improvement. For VR-12 MCS and VR-12 PCS, higher scores indicate improvement; therefore, negative Δ values indicate improvement. Signed Δ values are reported, rather than absolute changes. Bolding denotes statistical significance (*p* < 0.05).

**Table 4:** Baseline-adjusted regression output for PROMS analysis.

Outcome	Time Point	Model	Adjusted Obesity Effect	95% CI	*p*-Value	n
NDI	6-week	Linear regression	β = 3.6	−5.0 to 12.2	0.410	78
NDI	Final	Linear regression	β = 5.2	−4.9 to 15.3	0.310	33
VAS-Neck	6-week	Linear regression	β = 0.1	−0.8 to 1.0	0.830	78
VAS-Neck	Final	Linear regression	β = 0.2	−1.0 to 1.4	0.740	33
VAS-Arm	6-week	Linear regression	β = 1.7	−0.1 to 3.5	0.072	78
VAS-Arm	Final	Linear regression	β = 1.2	−1.0 to 3.4	0.280	32
PHQ-9	6-week	Linear regression	β = 1.7	−0.8 to 4.2	0.180	78
PHQ-9	Final	Linear regression	β = 2.1	−0.9 to 5.1	0.170	32
VR-12 MCS	6-week	Linear regression	β = −7.7	−14.6 to −0.8	**0.031**	78
VR-12 MCS	Final	Linear regression	β = −6.4	−12.6 to −0.2	**0.043**	28
VR-12 PCS	6-week	Linear regression	β = −4.1	−12.4 to 4.2	0.330	78
VR-12 PCS	Final	Linear regression	β = −6.0	−14.4 to 2.4	0.160	28
NDI MCID	Final	Logistic regression	OR = 0.92	0.22–3.85	0.910	33
VAS-Neck MCID	Final	Logistic regression	OR = 0.44	0.15–1.29	0.140	33
VAS-Arm MCID	Final	Logistic regression	OR = 0.80	0.18–3.48	0.770	32
PHQ-9 MCID	Final	Logistic regression	OR = 0.98	0.22–4.40	0.980	32
VR-12 MCS MCID	Final	Logistic regression	OR = 2.20	0.43–11.2	0.350	28
VR-12 PCS MCID	Final	Logistic regression	OR = 0.47	0.08–2.74	0.400	28

Linear regression models are used to adjust for BMI group, the corresponding baseline PROM, age, hypertension, diabetes, and CCI score. Logistic regression models are used for MCID achievement using the same covariates. β represents the adjusted difference between obese and non-obese groups; OR represents the adjusted odds ratio for MCID achievement. VAS-N = Visual Analog Scale-Neck Pain; VAS-A = VAS-Arm Pain; NDI = Neck Disability Index; PHQ-9 = 9-Item Patient Health Questionnaire; VR-12 MCS = Veterans RAND-12 Mental Composite Score; VR-12 PCS = Veterans RAND-12 Physical Composite Score. Bolding denotes statistical significance (*p* < 0.05).

## Data Availability

The data presented in this study are not available due to patient privacy restrictions.
